# Therapeutic applications of mesenchymal stem cells for amyotrophic lateral sclerosis

**DOI:** 10.1186/scrt421

**Published:** 2014-03-04

**Authors:** Christina M Lewis, Masatoshi Suzuki

**Affiliations:** 1 Department of Medical Physics, School of Medicine and Public Health, University of Wisconsin-Madison, 1111 Highland Avenue, Madison, WI 53705, USA; 2 Department of Comparative Biosciences, The Stem Cell and Regenerative Medicine Center, University of Wisconsin-Madison, 2015 Linden Drive, Madison, WI 53706, USA

## Abstract

Amyotrophic lateral sclerosis (ALS) is a neurodegenerative disease affecting the neuromuscular system and does not have a known singular cause. Genetic mutations, extracellular factors, non-neuronal support cells, and the immune system have all been shown to play varied roles in clinical and pathological disease progression. The therapeutic plasticity of mesenchymal stem cells (MSCs) may be well matched to this complex disease pathology, making MSCs strong candidates for cellular therapy in ALS. In this review, we summarize a variety of explored mechanisms by which MSCs play a role in ALS progression, including neuronal and non-neuronal cell replacement, trophic factor delivery, and modulation of the immune system. Currently relevant techniques for applying MSC therapy in ALS are discussed, focusing in particular on delivery route and cell source. We include examples from *in vitro*, preclinical, and clinical investigations to elucidate the remaining progress that must be made to understand and apply MSCs as a treatment for ALS.

## Introduction

Amyotrophic lateral sclerosis (ALS) is a rapidly progressing neurodegenerative disease characterized by the loss of upper and lower motor neurons (MNs). The mechanisms of cell death and functional deficits, and consequently the potential treatment approaches, are complex and varied. Cell therapy approaches complement this complexity well in their ability to respond to the host environment with multiple mechanisms of repair. Recently, new potentials of stem cells have been highlighted for the treatment of many human diseases. While various types of stem cells are available from different tissues, mesenchymal stem cells (MSCs) have been broadly applied as treatment to many disease types, including neurodegenerative diseases. In this review, we discuss the investigation of stem cell therapy using MSCs as a potential treatment for ALS. We describe the strengths of MSCs for cell therapy, the potential mechanisms of MSC actions in treating ALS, the design of MSC treatment and delivery, and the recent translation of this therapy from preclinical models into early-phase clinical trials.

## Amyotrophic lateral sclerosis

Neurodegenerative diseases are characterized by the progressive degeneration of selective neural populations with subsequent functional loss. ALS, also known as Lou Gehrig’s disease, is a fatal neurodegenerative disease caused by the selective loss of MNs in the spinal cord and brain stem. MN degeneration and neuromuscular junction (NMJ) denervation rapidly result in decreased motor function. In humans, death typically results 3 to 5 years after diagnosis because of respiratory failure after loss of diaphragm control. About 90% of ALS cases occur sporadically; the remaining 10% are familial (fALS). Approximately 70% to 80% of fALS cases have mutations of the Cn^2+^/Zn^2+^ superoxide dismutase 1 (SOD1), *TDP43*, *FUS*, or *C90ORF72* genes [1]. Rat and mouse models overexpressing mutated human SOD1 gene have been developed and follow patterns of pathology and disease progression similar to those observed in humans. These models are the basis for most *in vivo* preclinical research probing the causes of and potential treatments for ALS.

Although a disease cause of sporadic ALS has not been specified, this disease is generally regarded as resulting from factors involving environment, lifestyle, aging, and genetic predisposition [2]. Several proposed pathological mechanisms of disease include protein misfolding and aggregation, glutamate excitotoxicity, oxidative stress, mitochondrial dysfunction, glial cell activation and related inflammatory processes, and axonal transport defects [3]. Currently, the only available treatment approved by US Food and Drug Administration is riluzole, which has been shown to increase median survival in patients by about 3 months [4]. A treatment for ALS that more significantly slows disease progression and improves quality of life would drastically alter the prognosis for patients with this disease.

Owing largely to the modest effects and partly to minor concerns regarding side effects on the neuromuscular system [5], development of new and effective therapies has high priority and a variety of alternates are in various stages of development and clinical trial. These therapies include anti-glutamatergic, anti-oxidant, mitochondrial, and anti-inflammatory agents [2]. Gene therapy has been also explored for the delivery of supportive trophic factors. Recently, stem cell therapy has been of great interest for ALS treatment, particularly because of the potential for multiple mechanisms of action.

## Stem cell therapy

Cell therapy is a promising candidate for ALS treatment, largely because of the selective MN death and the variety of proposed mechanisms of degeneration that characterize the disease. The primary aim of stem cell therapy in neurodegenerative diseases is cell replacement, neuroprotection, or a combination of the two. Direct cell replacement may be challenging because of the anatomical and functional complexity of the central nervous system (CNS), whereas neuroprotection may be a more feasible short-term goal [6].

Multiple stem and progenitor cell types could have the potential to either directly replace MNs and diseased glia or provide support to slow degeneration. These cells include pluripotent cells such as embryonic stem (ES) cells and induced pluripotent stem (iPS) cells. ES and iPS cells are attractive in their potential for replacement of multiple cell types. Also, the establishment of a method for inducing pluripotency from adult cells reduces ethical issues surrounding the use of ES cells [7]. However, doubts remain about the functional potency of iPS cells, and these cells carry the risk of teratoma formation [8]. Tissue-specific progenitors, which are categorized as adult stem cells, are also candidates for cell therapy in neurodegenerative disease. These progenitor cells include neural progenitor cells and MSCs. These cells may be more accessible and more specific to the therapeutic target. Cell type selection for stem cell therapy must consider the likelihood of achievement of the intended goals of cell replacement or neuroprotection, along with availability, systemic effects on the host organism, and cost. Most importantly, the selected cell type must complement the intended therapeutic targets in each disease application. The therapeutic plasticity of MSCs matches the complex character of ALS well, making MSCs strong candidates for treatment of this disease.

MSCs are firstly identified as stromal cells from the bone marrow. These cells represent a small population of bone marrow cells and also have been identified in different mesenchymal tissues of fetal or adult origin. Morphologically, MSCs are mostly fusiform and fibroblast-like cells. The cells can be identified by negative and positive profiling of various hematopoietic surface markers, although differences exist among the reported studies in those surface marker characteristics [9]. The essential characteristic of MSCs is their ability to differentiate, either *in vivo* or *in vitro*, into bone, stroma, cartilage, ligament/tendon, and fat [10]. MSCs can be easily cultured to a large scale *in vitro* under appropriate conditions. MSCs have been known to express cytokines and growth factors such as transforming growth factor-beta, interleukin-10 and −6, insulin-like growth factor (IGF)-1, and vascular endothelial growth factor (VEGF), which are potentially involved in therapeutic contributions for neuronal protection and reduced inflammation following transplantation [11,12].

MSCs have been applied as treatment to many disease types, including neurodegenerative diseases. The safety of their clinical use has been established for treatment of hematopoietic disease. Treatment with human MSCs (hMSCs) is being explored and has reached phase I and II clinical trials in a variety of disease contexts, including graft-versus-host disease, heart disease, and several neurodegenerative disorders [13-15]. In these contexts, MSCs are recognized not only for their cell replacement capacity but also for their ability to respond to the needs of the host via secretion of supportive factors and modulation of immune response. The immunoregulatory capacity, potency, stability, and ease of harvest and expansion of MSCs make them attractive candidates for cell therapy for ALS. The delivery of these cells has been shown to slow disease progression, delay disease onset, and/or increase survival in preclinical models of ALS by a variety of mechanisms. These preclinical studies will be discussed further throughout this review.

## Therapeutic applications of mesenchymal stem cells for amyotrophic lateral sclerosis

**Figure 1:**
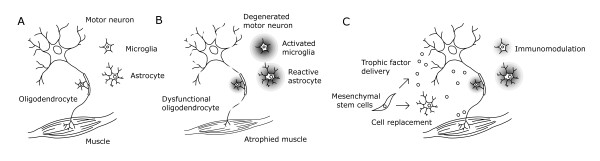
**Mechanisms of mesenchymal stem cell therapy in amyotrophic lateral sclerosis. (A)** In the healthy patient, motor neuron viability is maintained when supported by healthy astrocytes and oligodendrocytes. **(B)** In familial ALS, intrinsic and extrinsic factors contribute to the degeneration of motor neurons. These factors are not well understood and remain under investigation. **(C)** Mesenchymal stem cells are well suited to treat this complex disease because of their wide range of potential therapeutic responses, including direct cell replacement, trophic factor delivery, and immunomodulation.

Although more than a few studies have shown that MSC transplantation results in disease measure improvement, the exact mechanisms by which beneficial outcomes arise are not entirely understood. The roles that MSCs can play in the treatment of ALS are manifold . Several mechanisms of repair and support, including cell replacement, trophic factor or gene delivery, and immunomodulation have been either attempted or observed, sometimes in tandem [16]. These treatment mechanisms are illustrated in Figure 1. Although the most effective combination of mechanisms has not yet been established and optimized, the therapeutic plasticity of MSCs for ALS treatment is an asset of this approach [17].

### Cell replacement

Particularly for ALS, cell replacement therapy would be expected to achieve one or both of two potential aims. The first is to directly replace the degenerated MNs with new, functional ones. The second is to provide supportive glial cells to protect and support MNs. Cell replacement aims to provide a source of cells that will survive, home to the region affected by disease, differentiate into the intended cell type, and integrate into the surrounding neural circuitry [15]. If functional MN replacement were achieved, it would presumably restore function and ameliorate clinical symptoms. However, significant barriers to this endpoint must be overcome in the process, and astrocyte or oligodendrocyte replacement may be a more achievable goal.

The MN replacement approach will not be successful if there are extracellular factors contributing to the death or dysfunction of cells. Several proposed mechanisms of MN death in ALS, including glutamate excitotoxicity and oxidative stress, might affect transplanted along with endogenous neurons [18]. The temporal relationship between functional cell integration and disease progression must also be considered [19]. In this anatomical context, the process of homing, extending axons, and forming functional synapses has not been shown to be achievable within the time frame of ALS progression if cells are transplanted at a symptomatic stage [20,21]. Even if this can be achieved in humans, the question of whether MSCs can form functional neurons in the spinal cord remains unanswered.

Although the possibility of trans-differentiation of grafted MSCs into neuronal phenotypes has been demonstrated in some studies [22,23], their therapeutic contribution is still uncertain. Such studies have provided evidence of the ability of MSCs to differentiate into cells with neuron-like morphology, gene expression, and protein expression. Recent work by Park and colleagues [24] demonstrated a method for inducing differentiation of MN-like cells from hMSCs *in vitro*. However, this phenomenon is still controversial, particularly due to the lack of evidence of functional synapse formation between trans-differentiated MSCs. A recent *in vivo* study induced in MSCs overexpression of neurogenin 1(Ngn1), a transcriptional regulator that plays a role in initiating neuronal differentiation. It was found that transplantation of MSCs-Ngn1 improved MN survival in SOD1 mice but that transplantation of unprocessed MSCs did not [25]. Although no evidence that MSC-Ngn1 cells had differentiated into neuronal cells was presented, this study did show the increased ability of MSC-Ngn1 cells to migrate to the spinal cord compared to unmodified MSCs. Given the variety of challenges to replacement of MNs with MSCs, other strategies are likely to be more efficacious.

The delivery of glial cells for neuroprotection may be a more achievable goal. Although the loss of primary MNs is the hallmark of ALS, increasing evidence is pointing toward roles of dysfunctional glial cells in the disease process. A recent study demonstrated that astrocytes generated from tissue of patients with either familial or sporadic ALS were selectively toxic to MNs [26], and another found that astrocytes with the SOD1 mutation prompted neurodegeneration in healthy wild-type (WT) rodents [27]. Oligodendrocytes have also been shown to display abnormal patterns of degeneration and cell replacement in SOD1 mice [28]. In this study, the selective removal of mutant human SOD1 expression from oligodendrocytes in a mouse model delayed disease onset and increased survival. This provides some explanation for the results of several preclinical studies that found that WT glial cells and glial-restricted precursors have positive effects in treating ALS model rodents [29-31]. These studies suggest that cell replacement with healthy, WT astrocytes or oligodendrocytes derived from MSCs may slow degeneration. This type of cell replacement, aimed at neuroprotection, may be more functionally and pathologically feasible.

### Trophic factor delivery

The effectiveness of MSCs in improving ALS outcomes may be due in part to their secretion of neurotrophic factors, which play a variety of roles in normal neural function and particularly in neural repair. Trophic/growth factors are essential proteins for the maintenance, function, differentiation, and proliferation of neural cells. Supplementation with factors such as glial cell line-derived neurotrophic factor (GDNF), VEGF, ciliary neurotrophic factor, and IGF-1 is of interest as a possible treatment of ALS. Virus-based delivery of these growth factors has been shown to reduce MN vulnerability, promote cell survival, and improve clinical outcomes in SOD1 mice [32-34].

Similarly to viral vectors, MSCs can serve as effective and stable delivery vehicles. They express or can be stably transduced to overexpress trophic factors [23,35]. MSCs also migrate toward injury sites, which may allow more flexibility in treatment delivery when compared with viral vectors [36,37]. Part of the recovery mechanism by trophic factors may include endogenous restorative or regenerative processes such as induction of endogenous neurogenesis, gliogenesis, and synaptogenesis [38,39]. Neuroprotection, as a result of the reduction of apoptosis, reduction of demyelination, or an increase in astrocyte survival, is another possible mechanism of action of MSCs expressing neurotrophic factors [40,41].

Experimental studies have examined the effects of MSC-based trophic factor delivery on ALS disease progression, symptoms, and pathology. Recently, our group examined the beneficial role of hMSCs genetically modified to stably overexpress growth factors such as GDNF (hMSC-GDNF) and VEGF (hMSC-VEGF). Intramuscular transplantation of hMSC-GDNF significantly reduced NMJ denervation, whereas unprocessed hMSCs did not. hMSC-GDNF also protected large cholinergic MNs in the ventral horn and increased survival of SOD1 rats [12]. We also showed that hMSC-VEGF has positive effects on subject survival comparable to those of hMSC-GDNF. Furthermore, the combined delivery of GDNF and VEGF significantly slowed disease progression, reduced endplate denervation, and enhanced MN survival when compared with either of the growth factors delivered individually [42]. These studies show that the expression of one or more growth factors may improve the outcomes of MSC therapy by protecting MNs and maintaining MN endplates.

### Immunomodulation

Inflammation and immune response may play important roles in the pathological progression of ALS [43]. Changes in the morphology, number, and role of immune cells both outside of and within the CNS are observed both before symptom onset and throughout the disease [44]. Microglia, the immune cells of the CNS, may play both protective and toxic roles in ALS. The mechanisms of activation and action of glial cells at different disease stages are unclear. However, extensive evidence exists for complex, non-cell autonomous cascades of degeneration that ultimately contribute to MN damage [18].

MSCs may be well matched to these complicated patterns of inflammation, as they have been shown to play a variety of immunoregulatory roles (reviewed in [45]). MSCs reduce the proliferation of B cells, T cells, and natural killer cells and slow the maturation of dendritic cells. They also modulate immune cell function by reducing antibody production by B cells, reducing or inhibiting activation of dendritic cells and T cells, and reducing cytokine secretion by natural killer cells [46]. In the CNS, MSCs have been shown to migrate to areas of inflammation and reduce inflammation [47]. Several recent studies applying MSCs in experimental models of ALS have indicated attenuation of migroglial activation and reduction in reactive astrogliosis as potential mechanisms of improved clinical outcomes [21,48,49]. For these reasons, the immunomodulatory roles that MSCs play may be an added benefit of their use for cell therapy for ALS.

## Treatment strategy to design effective therapy

The technique and route of delivery of MSCs in ALS must also be considered in developing the most effective treatment approach [50]. Each potential mechanism discussed previously may be achievable in one or more anatomical compartments, and selection of the most efficient compartment is critical. Deliveries to the brain, spinal cord, intrathecal space, venous system, and skeletal muscle have all been investigated in preclinical models and may each be appropriate depending on the study design and intended mechanism of repair. The number of cell injections and time course of cell therapy administration can also affect cell survival and clinical outcomes. The major results from a selection of preclinical studies treating rodent models of ALS with MSCs are summarized in Table 1 for comparison of treatment techniques and key results.

**Table 1:** **Notable results from a selection of preclinical experiments treating SOD1**^**G93A **^**rodent models of amyotrophic lateral sclerosis with mesenchymal stem cells**

**Model and species**	**Cell type**	**Treatment route**	**Treatment timing**	**Clinical effects**	**Life-span effects**	**Post-mortem observations of MSCs and MNs**	**Other effects**	**Reference**
SOD1^G93A^ mouse	hMSC	Intraspinal (10^5^)	Pre-onset (week 28)	Significantly improved motor score and rotarod performance in treated males (week 32)	Not assessed	MSCs survived in spinal cord	36% decrease in number of CD11b^+^ microglia	Vercelli *et al*. [48] (2008)
						54% increase in MNs in treated females (week 38)	45% decrease in number of GFAP^+^ astrocytes (week 38)	
SOD1^G93A^ rat	rMSC	Intrathecal lumber spinal cord (2 × 10^6^)	Disease onset (week 13)	12% delay in paralysis onset	13% increase	MSCs survived in spinal cord 71% increase in number of lumbar MNs (week 18)	62% decrease in number of CD11b^+^ microglia (week 18)	Boucherie *et al*. [21] (2009)
SOD1^G93A^ mouse	hMSC from patient with ALS	Intrathecal (A) 10^4^	Pre-onset (week 8)	(A, B) No significant difference in motor performance	(A) No significant change	MSCs detected in ventricular system, subarachnoid space, brain, spinal cord	No significant difference in disease onset	Kim *et al*. [54] (2010)
		(B) 2 × 10^5^						
					(B) 4.7% increase			
		(C) 10^6^						
				(C) Significantly delayed decline in rotarod performance	(C) 6.5% increase			
						(A) No significant change in number of MNs		
						(B) 41% increase in		
						number of MNs		
						(C) 79% increase in number of MNs (week 15)		
SOD1^G93A^ mouse	Encapsulated hMSC-GLP1	Intracerebro-ventricular	Pre-onset (week 5)	Significantly delayed disease onset and weight loss	11% increase	Capsules not detected		Knippenberg *et al*. [55] (2012)
		(2.5-3 × 10^3^)						
						No significant change in MN count		
				Significantly delayed decline in rotarod performance				
SOD1^G93A^ rat	rMSC	Intraspinal (10^5^) and intravenous (2 × 10^6^)	Disease onset (week 16)	Significant BBB test and grip strength difference starting 4 weeks post-injection	6.1% increase	MSCs survived in spinal cord		Forostyak *et al*. [20] (2011)
						55% increase in number of thoracic MNs		
						37% increase in number of lumbar MNs (end stage)		
SOD1^G93A^ mouse (irradiated)	hMSC	Intravenous (3 × 10^6^)	Pre-onset	9.0% delay in disease onset	9.8% increase	MSCs detected in brain, brainstem, and spinal cord throughout disease progression		Zhao *et al*. [60] (2007)
			(week 8)					
				3-week delayed decline in rotarod performance				
						23% increase in number of lumbar MNs (week 16)		
						45% increase in number of lumbar MNs (week 20)		
SOD1^G93A^ mouse	(A) hMSC	Intravenous (10^6^)	Pre-onset (week 8)	(2B, 3B) Improved motor performance week 16	(3B) 7.3% increase	(1A, 1B) MSCs detected in spinal cord (week 10) (1A) No change in MN number (1B) 57%		Chan-Il *et al*. [25] (2013)
					(1A, 1B, 2A, 2B, 3A) No significant effect			
						increase in number of cervical MNs, 50% increase in number of lumbar MNs (week 16)		
	(B) hMSC-Ngn1							
			Disease onset (week 14–16)					
			Disease onset (weeks 13 and 15)					
SOD1^G93A^ mouse	mMSC	Intravenous (10^6^)	Disease onset (week 12)	Significantly delayed decline in motor performance (rotarod, extension reflex, gait impairment)	15% increase	MSCs detected in spinal cord at 24–48 hours with decreasing numbers over time	24% decrease in ubiquitin^+^ cells	Uccelli *et al*. [61] (2012)
							16% decrease in GFAP^+^ astrocytes	
							34% decrease in IB4^+^ microglia	
						No significant change in MN count		
							(spinal cord, week 17)	
				Significantly increased body weight (week 16 onward)				
SOD1^G93A^ rat	(A) hMSC	Intramuscular (1.2 × 10^6^ per time point)	Pre-onset once/week for 3 weeks	(A, B) Significantly slower motor dysfunction progression (measured by BBB test score)	(A) No significant change	MSCs survived in muscle	(A) No significant effect on NMJ innervation or denervation	Suzuki *et al*. [12] (2008)
	(B) hMSC-GDNF							
					(B) 17% increase	(A) 28% increase in number of ChAT^+^ lumbar MNs		
			(weeks 11–13)					
							(B) Significantly Increased NMJ innervation and decreased denervation (week 17)	
						(B) 36% increase in number of ChAT^+^ lumbar MNs (week 17)		
SOD1^G93A^ Rat	(A) hMSC	Intramuscular (1.5 × 10^6^ per time point)	Pre-onset once/week for 3 weeks	(C, F) 5% delay in disease onset	(B) 10% increase	(A-F) MSCs survived in muscle	(B, C, F) Significantly increased NMJ innervation	Krakora *et al.*, 2013 [42]
	(B) hMSC-GDNF							
	(C) hMSC-VEGF			(A, B, D, E) No significant effect on onset	(C) 7.5% increase	(A) No significant change in number of large lumbar MNs		
			(weeks 12–14)					
							(F) Significantly increase NMJ innervation compared with (B, C) (week 21)	
	(D) hMSC-IGF-1							
					(A, D, E) No significant change			
				(F) Significantly slower motor dysfunction progression (measured by BBB test score)				
	(E) hMSC-BDNF							
					(F) 16% increase	(B) 200% increase in number of large MNs		
						(C) 150% increase in number of large MNs		
	(F) hMSC-GDNF/VEGF							
						(F) 230% increase in number of large MNs (week 21)		

All listed percentage changes were found to be significant by the original authors. Changes are compared with sham, vehicle control, or untreated subjects unless otherwise noted. ALS, amyotrophic lateral sclerosis; BBB, Basso Beattie Bresnahan; BDNF, brain-derived neurotrophic factor; ChAT, choline acetyltransferase; GDNF, glial cell line-derived neurotrophic factor; GFAP, glial fibrillary acidic protein; GLP1, glucagon-like peptide 1; hMSC, human mesenchymal stem cell; IGF, insulin-like growth factor; mMSC, mouse mesenchymal stem cell; MN, motor neuron; MSC, mesenchymal stem cell; Ngn1, neurogenin 1; NMJ, neuromuscular junction; rMSC, rat mesenchymal stem cell; VEGF, vascular endothelial growth factor.

### Direct delivery into the central nervous system

Direct delivery of MSCs to the spinal cord, brain, or intrathecal space offers an efficient administration route to the diseased areas and may reduce the volume of treatment agent necessary. However, it may involve more clinical complications because of the need for CNS surgery. The proximity of the therapeutic agent to its target may maximize the likelihood of cell replacement within the time course of disease progression. Cell delivery to regions of the spinal cord involved in limb and respiratory function is of particular interest, as loss of respiratory function is the cause of death in most cases of human ALS.

Several preclinical studies and most clinical trials of MSC therapy in ALS have focused on delivery to the spinal cord [20,48,51-53]. Even though a small number of MSCs were transplanted into the lumbar spinal cord, they migrated well to the ventral horn and were located in close proximity to MNs. An increase in MN number and delay in motor function loss were observed [48]. Although spinal delivery has not resulted in significant safety issues in clinical studies, concerns regarding potential surgical damage to the diseased area and the increasing evidence for non-replacement mechanisms of MSC repair have prompted the investigation of other delivery routes.

Intracerebroventricular and intrathecal deliveries avoid direct intervention in the affected regions of the spinal cord. Preclinical studies have shown similar effects when compared with spinal transplantation, including increased survival, delayed disease onset, and increased MN survival in treated subjects [54,55]. Safety of these delivery methods has also been indicated in clinical trials [56-58].

### Systemic or intramuscular delivery

The delivery of MSCs to tissues outside the CNS may avoid complications related to surgery and could take advantage of the plasticity of MSCs as therapeutic agents. However, it may be less efficient than CNS delivery. It is expected that indirect delivery will require greater doses for results comparable to those of direct delivery, but this has not been established conclusively. There is growing evidence for systemic and non-cell autonomous mechanisms of neurodegeneration in ALS; this evidence suggests that treatments delivered outside the CNS may be appropriate [46,59].

Intravenous delivery of MSCs has been shown to significantly improve disease measures in rodent ALS models. In preclinical studies, MSCs administered intravenously into SOD1 mice resulted in cell migration to various peripheral tissues as well as to the brain and spinal cord [25,60-62]. Significant increase in life span and delay of disease progression were observed in two of these studies [60,61], and Uccelli and colleagues [61] suggest reduced microglia activation and reduced oxidative stress as potential mechanisms of repair. A recent clinical study found no significant safety issues with this delivery method and reported statistically significant decreases in lymphocyte number, dendritic cells, and lymphocyte proliferation after intravenous administration [56].

Cell delivery to the muscle has also been proposed with the aims of maintaining and protecting the NMJ. In preclinical studies, MSC therapy via intramuscular injection has been shown to reduce NMJ denervation, improve MN survival, increase survival, and be an effective trophic factor delivery technique in SOD1 rodents [12,42]. Intramuscular delivery has the advantage of proximity to the affected area without requiring direct intervention into the CNS.

### Delivery dose and time course

A challenge in comparing cell therapy techniques and optimizing delivery strategy is the variety of approaches that researchers employ with respect to number of injection sites, number of transplantation time points, and cell dose, as shown in Table 1. Some researchers adopt a multiple-transplant approach to improve cell survival, whereas others deliver cells to multiple locations or with multiple delivery routes [12,20,63]. These strategies may allow an increased total cell dose while avoiding safety complications that could result from one large administration to a single location. Although phase I clinical trials will help to determine safe cell doses, preclinical studies directly comparing success of different treatment approaches would aid in the selection of approaches for clinical testing.

### Cell source: autologous versus allogeneic

One of the advantages of MSCs in comparison with other stem cell types is the relative ease of harvest and *ex vivo* expansion, resulting in the possibility of autologous stem cell transplantation. This approach may increase the safety of the technique in avoiding issues of immune rejection while avoiding political and ethical issues regarding stem cells derived from fetal sources [15]. However, the patient-derived cells may have reduced or altered therapeutic effects. Several recent *in vitro* and *in vivo* studies suggest that MSCs and other stem cells derived from patients with ALS may have reduced pluripotency, hampered trophic factor secretion efficiency, and toxic effects on MNs, particularly if harvested at a later disease state [26,27,64,65].

These studies encourage further investigation into the relative effects of autologous and allogeneic MSCs for treating ALS. Most studies in experimental models have used allogeneic MSCs with encouraging results. The use of these cells is more affordable and, owing to the immunosuppressive roles that MSCs play, may not require immunosuppression (and have even been used as a treatment to graft-versus-host disease) [16].

## Current clinical trials using mesenchymal stem cells for amyotrophic lateral sclerosis

There have been several early-phase clinical trials of the treatment of ALS with hMSCs [51,56,57,66]. Bone marrow cells (BMCs), which may consist of mesenchymal and hematopoietic stem cell types, have also been investigated for cell therapy in ALS [52,53,67]. The primary goal of these studies was to establish the safety of the therapeutic agent and delivery method. In all of these studies, autologous MSCs or BMCs were isolated from patients, characterized, and used as treatment. Four of these studies have delivered cells intraspinally and found no significant safety issues related to the implantation procedure [51-53,66]. In one of these studies, histology performed on three patients revealed that the MN number near the level of the graft (4.2 ± 0.8 MNs per section at T4-T5) was significantly higher than that farther from the graft (0.9 ± 0.3 MNs per section at T1, T8-T9) and did not vary significantly between patients [53]. No significant changes in disease progression were recorded, although researchers reported anecdotal evidence of improvement of symptoms. In two studies, cells were delivered intrathecally or intravenously [56,57]. These report similar results with respect to safety and response. One study that performed immunological evaluation 24 hours after cell engraftment reported significant reduction in activated lymphocytes and cytokine production, indicating immediate immunomodulatory effects of the hMSCs [56]. One study delivered stem cells to the motor cortex; this delivery method involved more safety complications [67].

Several proposed and ongoing clinical trials focus on intrathecal delivery (National Institutes of Health ClinicalTrials.gov identifiers NCT01082653 and NCT01142856), and one explores intramuscular delivery and the delivery of unspecified neurotrophic factors (NCT01051882). Once the safety of all delivery methods is established, information from human studies determining optimal cell dose and from experimental studies determining the most efficient and effective treatment method can be combined to optimize the clinical application of hMSCs in ALS.

## Conclusions

In the past decade, great accomplishments have been made in the development and clinical translation of stem cell therapies for ALS. MSCs stand out as cells capable of protecting MNs, differentiating into multiple neural cell types, modulating immune cell roles, and reducing CNS inflammation. The success of MSCs in delaying disease onset, improving motor function, and increasing survival in preclinical models of ALS has resulted in multiple clinical trials of MSC therapy in patients with ALS. These trials have established the safety of MSC delivery for CNS applications, opening the door for larger late-phase trials to better understand the effectiveness of MSC therapy in humans. With this in mind, further work is necessary to maximize the potential of MSC therapy for ALS.

The establishment of the most effective technique for delivering MSCs to patients is essential. As treatment techniques in preclinical studies vary greatly between groups of investigators, within-group studies comparing multiple techniques may help elucidate the optimal approach. In particular, studies comparing delivery techniques alone or in combination, and investigating resulting mechanisms of repair will clarify the most important roles that MSCs play in treating ALS. *In vivo* cell-tracking techniques are essential to aid these studies and elucidate the initial distribution, migration, and survival of engrafted cells.

Further studies examining the effectiveness of MSCs overexpressing various growth factors to reduce MN degeneration and improve clinical outcomes will help to maximize the positive effects of cell therapy. In particular, overexpression of anti-inflammatory factors or those that prevent misfolding and aggregation of proteins may also be worthy of investigation. In this way, researchers can, and should, take advantage of the therapeutic plasticity of MSCs. All potential reparative roles of MSCs post-delivery, many of which are described above, should be carefully considered and incorporated to maximize the effectiveness of MSC therapy in ALS.

## Abbreviations

ALS: Amyotrophic lateral sclerosis; BMC: Bone marrow cell; CNS: Central nervous system; ES: Embryonic stem; fALS: Familial amyotrophic lateral sclerosis; GDNF: Glial cell line-derived neurotrophic factor; hMSC: Human mesenchymal stem cell; IGF: Insulin-like growth factor; iPS: Induced pluripotent stem; MN: Motor neuron; MSC: Mesenchymal stem cell; Ngn1: Neurogenin 1; NMJ: Neuromuscular junction; SOD1: Superoxide dismutase 1; VEGF: Vascular endothelial growth factor; WT: Wild-type.

## Competing interests

The authors declare that they have no competing interests.
